# Progress in Surgery

**Published:** 1902-02-08

**Authors:** 


### Progress in Surgery.

OTOLOGY.—*Continued from page* 308.

**Sclerosis of the Middle Ear.**—Mayo Collier[6] has contributed a paper on the causation and treatment of this affection. He has arrived at the conclusion that a very large percentage of the cases are a concomitant and direct result of ordinary nasal catarrh from simple mechanical obstruction to the Eustachian tube. The tympanic cavity and the other cavities leading from the nose are anatomically extensions from the nose cavity proper and share in the diseases of the latter. Some of the writers' opinions contrast sharply with those of Cobb quoted above. Thus Collier writes: "The idea that chronic non-suppurative disease of the middle ear is always due to an extension of a catarrhal or inflammatory process from the nose or naso-pharynx is entirely unwarrantable. The influence exerted by the inflammatory condition of the nose or naso-pharynx is usually purely mechanical," and again later he contends "that chronic non-suppurative disease of the middle ear in the majority of cases is due to the mechanical obstruction to the Eustachian tube." Cobb, on the other hand, speaks of the harmlessness of nasal obstruction. Collier believes that in chronic cold in the head with partial obstruction to nasal respiration there is present every factor requisite to produce hypertrophy and hyperplasia, or sclerosis of the middle ear. One-quarter to one-third of all ear diseases are of this nature, and its absolute frequency in the total population is stated to be very great. The ages of the subjects of the affection vary from 5 to 60, but, as S. Seaton pointed out, most of the cases develop between 20 and 30, and the next most prolific decade is between 10 and 20. The writer suggests that many cases of what he would call "latent obstruction to the nose are missed because examined when the patient is up and about, and gravity has reduced the swollen condition of the interior, rendering the nose patent by day, but leaving it obstructed when the individual is in the horizontal position." Future progress in treatment is not to be looked for in operations on the membrana tympani. The early recognition of the affection and use of a nasal wash and Politzer's bag will go a long way in restoring the patient to health.

**The Treatment of Otorrhœa.**—D. W. Aitken[7] advocates a method of treatment useful in acute cases, but still more useful in chronic cases in which the mastoid has become affected. First some of the lotion selected is poured into the ear. Then a plug of wadding large enough to fit the meatus is fixed upon a probe—a probe with two spiral teeth at the end is the best, as it holds the wool firmly and yet allows it to be removed easily by rotating the stem counter-clockwise. The probe and wool are then used like the piston in a syringe and gently pushed inwards and outwards. The fluid is thus made to reach both the attic and the mastoid recesses. Much discharge can be brought to the surface by this procedure even after syringing and swabbing have been efficiently performed. After some repetition the lotion will return clean. Then any medicament can be applied to the clean surfaces. It is recommended to begin with chinosol, iodoform, or amyloform in absolute alcohol. The application of absolute alcohol is practically painless in almost all cases, and in the others it causes only momentary smarting. Its advantages are that (1) it acts promptly on polypoid growths; (2) it is a most satisfactory antiseptic; and (3) as it evaporates it leaves a dry surface. When the solution has been poured into the ear the "piston-rod" is used several times, and thus the fluid is forced into all recesses.

**Aural Polypi.**—H. F. Waterhouse[8] has discussed these growths. He alludes to the three varieties of polypi, the mucous, fibrous, and myxomatous. More than 80 per cent. belong to the mucous variety, and more than 90 per cent. originate in the mucous lining of the tympanum. Polypi tend to dam back the flow of pus in the ear. They are attended at times with discharge of blood mixed with fœtid pus, with neuralgic pains, tinnitus, giddiness, and attacks of vomiting. Not much importance is attached to the differentiation between polypi and granulations. The latter are commonly found in connection with caries and necrosis of the tympanic walls. Aural polypi, the writer points out, constitute a serious disease, and may occasion fatal troubles, and the treatment, *i.e.* their removal, is to be regarded as a somewhat hazardous procedure. In certain cases, when the bony roof of the tympanum is eroded or congenitally incomplete, the polypus may practically spring from the dura mater, and provide a path by which pyogenic organisms may gain access to the interior of the cranial cavity. Waterhouse recommends as a preparatory treatment before removal, that a solution of biniodide of mercury, 1 to 3,000 in rectified spirit, be introduced into the ear three times daily. This antiseptic solution penetrates the tissues well, as it does not coagulate albuminious discharges, and probably more importantly dehydrates the polypus. After removal by Wilde's snare, or by twisting with Toynbee's forceps, the root of the polypus must be destroyed with the electric cautery or with a tiny bead of chromic acid. When removed, fatal absorption may take place through the open mouths of the vessels in the base of the tumour. After removal, the biniodide lotion should be continued. Almost complete anæsthesia for the removal of polypi may be obtained by using the solution recommended by Dr. Gray, viz., a 10 per cent. solution of cocaine in equal parts of anilin oil and rectified spirit.

**Foreign Bodies in the Ear.**—P. Fridenberg (New York)[9] has written on this subject. Unless the body be lightly impacted, or presses on the drumhead, or is of an irritating nature, its presence may cause very slight discomfort, and there is no need of hurry in its removal, and care has to be taken that remedial efforts do not produce worse results than the bodies themselves. Politzer has said that "the consequences attributed to the foreign bodies are, with rare exceptions, provoked by unskilful attempts at extraction, undertaken by unpractised hands." The affection is not common. The presence of the body may be discovered quite accidentally, and the patients may not come up for treatment until their hearing has been interfered with for years. The severity of the symptoms depends chiefly on the degree of force with which the body has entered and the depth to which it has penetrated. Some of the symptoms are slight deafness, ringing in the ears, and autophony. Very rarely reflex symptoms, such as vomiting, hiccough, neuralgia, paræsthesia, facial tic, and even epileptiform seizures may arise. Foreign bodies may be overlooked (1) if the body has penetrated the drum and passed into the tympanum; (2) if the canal has become swollen from the injury inflicted; (3) if the body is incorporated with cerumen; and (4) when the sinus of the auditory canal is very deep and the body lies in it. The sinus is a hollow in the floor of the meatus close to the drumhead. Syringing is the best method of removal, but it may be contra-indicated by the presence of a large perforation of the drum, the development of nausea, or if the body is sharp pointed and likely to wound the drum. A mixture of alcohol and ether has been advised for instillation if the mass is swollen by imbibition of moisture, but hot water will usually suffice. The following methods are mentioned as "therapeutic curiosities": the use of a small camel's-hair paint-brush dipped in glue and allowed to fix itself to the body and then withdrawn; the removal of the body by means of aspiration with an ear syringe attached to a rubber tube; and irrigation by a catheter from the nose. If instruments are used such as hooks, spoons, wire loops, forceps, or snares, they must only be applied under the guidance of vision. If all ordinary attempts fail the body may have to be exposed by an operation in which the pinna is deflected forwards and the cartilaginous meatus divided.

[6] Brit. Med. Jour., Sept. 28, 1901, p. 894. [7] Vide abst. Jour. L. R. and O., July 1901, p. 373. [8] Edin. Med. Jour., Sept. 1901, p. 223. [9] Med. Rec., Sept. 21, 1901, p. 447.

